# Relationship Between Meaningful Activity Engagement and Well-Being Moderated by Race

**DOI:** 10.1093/geroni/igab046.3164

**Published:** 2021-12-17

**Authors:** Dugan O'Connor, Jennifer Smith

**Affiliations:** Mather, Evanston, Illinois, United States

## Abstract

Engaging in meaningful activities has benefits for health and well-being in older adults; however, racial differences in meaning from activities have been observed. The current study examined how the relationship between engagement in a variety of meaningful activities and well-being differs for Black and White older adults. Participants (130 White and 101 Black older adults), ages 53 to 90 (M=69), completed an online survey that measured the extent to which engaging in various activities provided meaning or fulfillment as well as measures of purpose in life, positive affect, negative affect, and loneliness. Overall, the most meaningful activities included “spending time with family,” “reading,” and “being outdoors/experiencing nature.” The least meaningful activities included “job/career,” “caring for pets/animals,” and “supporting a social or political cause.” Black older adults reported greater meaning from “religious faith,” “spiritual practices/meditation,” “spending time with family,” and “listening to music,” and less meaning from “caring for pets/animals,” compared to White older adults. A series of multiple regression analyses (with age, gender, education, and income as covariates) revealed that greater meaning across activities was associated with lower loneliness, regardless of race. Significant Meaning x Race interactions revealed meaning was positively associated with sense of purpose and positive affect for Black but not White older adults. These findings suggest that finding meaning in leisure activities is a psychological resource that may contribute to Black older adults’ well-being. The racial differences in sources of meaning further support the importance of considering the target population when designing activity programs for older adults.

